# Teamwork and use of teams in services for older people: a qualitative study of finnish nurse managers’ experiences

**DOI:** 10.1186/s12912-025-03418-w

**Published:** 2025-07-01

**Authors:** Henrika Karhulahti-Nordström, Visa Väisänen, Saukkonen Petra, Alastalo Hanna, Timo Sinervo

**Affiliations:** 1 Finnish Institute for Health and Welfare, Welfare State Research and Reform Unit, Health and Social Service System Research Team, Mannerheimintie 166, Helsinki, 00330 Finland; 2 Department of Health and Social Management, Faculty of Social Sciences and Business Studies, University of Eastern Finland, Yliopistonranta 8 E, Kuopio, 70210 Finland; 3 Finnish Institute for Health and Welfare, Services Unit, Older People Services Team, Mannerheimintie 166, Helsinki, 00330 Finland

**Keywords:** Home care, Assisted living with 24/7 services, 24-hour care, Teams, Teamwork, Workforce planning, Continuity of care, Services for older people

## Abstract

**Background:**

In Finnish services for older people, teams (a permanent group with a common task, divided for example by geographical or spatial criteria) have been implemented with the goal of improving the quality of care, individualized care and continuity of care as well as the wellbeing of nurses. Our aim was to describe nurse managers’ experiences of using teams in home care and assisted living with 24/7 services.

**Methods:**

The qualitative data used in the study was collected in May 2023 as part of a national survey on care organization for all units (*n* = 2996) offering care services for older people in Finland. Nurse managers working in home care and nurse managers working in assisted living with 24/7 services were asked to describe the benefits and challenges they have faced with the use of teams.

**Results:**

Nurse managers highlighted that using teams enabled better planning and evaluation of client care, nurses’ better wellbeing and ability to cope with work demands, and higher quality of care. Perceived challenges were incorporating temporary workforce into teams, the frequent poor collaboration between different teams, and perceived hindrance of professional development.

**Conclusions:**

Teamwork might be beneficial for promoting wellbeing at work and for reducing the psychosocial burden experienced by nurses as well as increasing both the quality and continuity of care. Maintaining and developing the professional skills of nurses needs to be ensured, and adequate collaboration between the teams is required, especially with the rapid increase in the use of temporary workforce.

## Introduction

As the population ages, the need for services and nursing staff increases. At the same time, there is a need to improve the quality of client work in services for older people [1]. Nurses are increasingly leaving the field entirely and staff turnover remains high [2, 3]. Developing nurses’ well-being at work and reducing the willingness to change jobs is essential for the client’s continuity of care and the quality of care [4, 5]. In connection with nurses’ career changes and intentions to change jobs, the issue of salaries and benefits rises in public debate. However, nurses’ job satisfaction has also been found to be a significant factor linked to intentions to change jobs [6]. According to previous research, job satisfaction is affected by psychosocial workload factors such as time pressure and limited opportunities to influence work [7], inadequate management [3], poor work organization [8], and workplace abuse [9]. A study by Ring and colleagues [10] on home care workers reasons for leaving work highlighted a mismatch between needs and resources, high ethical burden, and not having the opportunity to carry out their work as they wished.

In services for older people, self-organizing teams have been implemented with the aim of improving the quality, individuality, and continuity of care as well as the wellbeing of employees. Self-organizing teams also aim to respond to the issues of increased care and workforce needs [11, 12]. Previously, self-organizing teams have been found to reduce workload, increase job satisfaction and better engage employees [13, 14]. In addition, the use of self-organizing teams is also reflected in client satisfaction, as clients have experienced better availability of services and continuity of care [15, 16]. Without constant staff, the continuity of care can be, however, impaired if the client is constantly visited by different care professionals [17].

The Buurtzorg model [18, 19], developed in the Netherlands, consists of independent home care teams of twelve nurses who are responsible for the comprehensive care of their clients and independently plan and make decisions related to the client’s care. High continuity of care and client-centered care are central to the model. Organizations lack middle management, and the nurses are allowed to independently organize and manage their own work, including holidays and recruitment. If necessary, the teams have the support of a coach in case of problems.

There are also challenges associated with self-organizing teams, as autonomy in teams does not arise by itself but requires resources [12, 14]. Self-organizing teams are challenged by staff turnover and the use of substitute and temporary workers [11]. As such, the introduction of self-organizing teams requires a change in organizational culture and leadership style [20]. Promoting self-organizing requires that the management is able and willing to support the employees. As resolving potential conflicts has been found challenging in self-organized teams [21, 22], it is important to pay particular attention to the interaction between management and employees [23]. Furthermore, the leadership should adapt a coaching direction to enable nurses to take sufficient responsibility for their work [12]. Ruotsalainen and colleagues [14] found that leaders of healthcare organizations should give teams real autonomy in their work so that the team can operate in a fully self-organizing way.

Self-organized teams emphasize autonomy and decision making of nurses. In most models of work and organization psychology autonomy and possibility of using skills are seen as positive factors in regard to lower stress levels, higher job satisfaction, motivation and job performance. According to Karasek’s Job Demands-Resources (JDC) model [24], excessively high job demands, in relation to too few opportunities to influence the work, and low possibilities to use skills lead to excess strain on employees. High job demands and high job control (high autonomy and possibilities to use skills), in turn, are supposed to lead to low stress, high motivation and learning at work [25]. Bakker & Demerouti’s JD-R model [26], on the other hand, looks at the issue from a broader perspective. When examining job requirements and resources, related factors, such as autonomy, feedback, coaching leadership, quality of teamwork, and other organizational, social, and individual resources, are considered. These factors are seen to be linked to employee motivation and workload, which in turn are associated with employees’ perceived wellbeing at work and quality of care.

In this article, autonomy refers to a nurse’s decision-making ability based on her professional knowledge base [27]. This does not necessarily mean that fully self-directed teams are needed to improve employee satisfaction and commitment. Improved job satisfaction could also be achieved by combining different autonomy practices [28]. Autonomy as a concept is multidimensional, involving both clinical and professional autonomy [29, 30]. While a lack of autonomy has been found to prevent nurses from working effectively [31], it should be noted that autonomy alone does not guarantee the success of self-leadership. Self-leadership could be supported through training or by fostering a work environment that supports autonomy. Importantly, autonomy of teams does not automatically mean autonomy of individual workers [32, 33].

In Finland, services for the older people are organized by 21 wellbeing services counties (and the city of Helsinki) and provided by the counties and private firms. It consists mainly of home care and assisted living with 24/7 services. Assisted living with only day-time services has been available only by clients’ own expense but is now increasingly coming to the formal service system, too. The current policy aim is enabling older people to live at home for as long as possible. [34] In 2023, 14% of persons aged 75 or over received regular home care. As clients’ need for services grows, clients are mainly cared for in assisted living with 24/7 services. In 2023, 7% of those aged 75 or over were residents in assisted living with 24/7 services [35]. 

In home care and assisted living with 24/7 services, the largest occupational group is practical nurses, who accounted for around 70% of employees in 2023. Registered nurses comprised 11% of the workforce in home care and 7% in assisted living with 24/7 services, respectively [35]. Practical nurses have completed a three-year vocational education, and their work mainly consists of assisting clients in their daily activities. The work of registered nurses emphasizes medical procedures, even though they also assist clients in daily activities [17]. 

When planning and developing care services for older people, the consequences on the wellbeing and retention of nurses should be further considered. Currently, personnel impacts are not sufficiently taken into account when developing services for the older people [36]. However, the importance of promoting wellbeing at work has been found to be central in ensuring the availability and commitment of nurses [5, 37]. In addition, alternative working arrangements and changes in work culture have helped attract and retain staff [12]. Management also needs to be examined, as the leadership style of nurse managers can have a significant impact on nurses’ perceived wellbeing at work [38].

Self-organized teamwork has developed also in Finland, with the aim of increasing the quality of care and the well-being of employees. Self-management of the teams has been found to have a positive impact on the employees’ perceived well-being at work and the quality of care in home care and assisted living in Finland. In home care, the results are not as clear, which may be since in assisted living the teams work together continuously, while in home care the work is usually more independent with the nurses mainly working alone [11, 5].

The Buurtzorg model as such is not fully used in Finland, although the model has been used in developing teamwork [11]. This is partly due to the differences between the Finnish and Dutch care systems, including legislation. Similar observations have been made in other countries where the Buurtzorg model has been tested [15, 39]. In Finland, home care and assisted living organizations can decide whether they use teams, and what the definition of a team is in their organization A team can be part of an official organizational structure and have its own fixed staff. The unit’s teams usually have 1–2 nurses appointed as team leaders, who take care of the day-to-day management of the teams. In most cases, however, the unit’s supervisor has administrative responsibility for the teams. In this study, teams refer to working in a permanent group or team with a common task.

Teamwork has been defined in many ways in the literature, and the definitions vary according to the context. While the use of teams is relatively widespread, information about nurse managers’ views and experiences of teams in home care and assisted living with 24/7 services is scarce. Assisted living with 24/7 services and home care also differ from each other in that employees in home care do not, as a rule, have the immediate support of colleagues all the time. In this context, it is also important to find out whether managers’ views differ on teamwork between assisted living with 24/7 services living and home care. Consequently, the aim of this study was to describe the nurse managers’ experiences (benefits and challenges) of using teams in home care and assisted living with 24/7 services. The following research question was formulated: What benefits and challenges has using teams brought to your unit?

## Methods

The study was carried out as a descriptive qualitative study to highlight the experiences with using teams working in home care and assisted living with 24/7 services. We used two qualitative questions from national survey data collected separately by the Finnish Institute for Health and Welfare (THL). The survey was conducted in May 2023 for all units organizing care services for older people (*n* = 2996). The survey, carried out every second year, is mandated by law and has been developed for administrative purposes, and for this study we have obtained a data permit to access the questions related to teamwork and the background information of the units (unit type). The used survey has been published separately in a report by Kehusmaa and colleagues [40].

The response rate for the entire survey was 92%. First, a question “Is the nursing staff of your unit divided into smaller teams?” was asked. If an affirmative answer was given, the following optional open-ended question was presented: “What benefits and challenges has using teams brought to your unit?“. In total, 449 nursing managers in home care and 713 in assisted living with 24/7 services indicated that they used teams, of which 355 nurse managers in home care and 555 in assisted living with 24/7 services answered the open-ended question, resulting in a response rate of 79% in home care and 78% in 24-hour assisted living.

In the survey, a team was defined as a permanent group or a team with a common task, which could be divided for example into geographical (e.g. district/block) or spatial (e.g. wing/corridor) criteria. Respondents were asked to answer questions about the teams, although the term “team” was used to refer to the division of the unit. If the unit was divided into several levels (the unit is divided into smaller entities that are further divided into smaller teams), respondents were asked to answer questions about the teams with the lowest team level in mind. It is important to note that teams in this context can refer to both regular teams of varying team models and self-organizing teams. In the study, we were unable to distinguish between different kinds of teams.

The answers were analyzed using data-oriented thematic analysis to highlight the experiences of the participants [41, 42]. First, one researcher (HK-N) read through the material five times to gain an overall understanding. Second, HK-N independently coded the responses based on the research question, with home care and assisted living with 24/7 services analyzed separately. Original expressions related to the benefits and challenges of using teams experienced by nursing managers were extracted from the answers. A word or sentence was chosen as the unit of analysis, as the responses ranged from single words to four sentences. The units of analysis (*n* = 2265) were then coded.

The codes were developed in a data-driven manner and iteratively refined as the analysis progressed. Coding was conducted using ATLAS.ti 24 programme. If the original expression was a single word, it was used as the code; if it was a sentence or multiple sentences, an abbreviation of the original expression was used. The code names were chosen to match the original expressions as closely as possible. After coding, the codes were organized into code groups and transferred to Excel, where they were further organized by group. Groups of codes with similar content were clustered into subcategories (*n* = 42). These subcategories were then combined into generic categories (*n* = 54), which were further grouped into two main categories: the benefits of using teams and the challenges of using teams (Table 1). Subgategories were given a descriptive name for the content. The initial codes with same content were grouped and named as subcategories (*n* = 54) and further as main categories (*n* = 2) (Table 1).

Another researcher (TS) independently reviewed the coding and classifications. The results were compared, and any discrepancies were discussed in joint meetings (HK-N, TS, VV). The upper and main categories were formed by combining similar codes and examining their relationships, internal consistency, and relevance to the research question. The final categories were refined through three rounds of analysis and compared with the original data to ensure credibility. The analysis was concluded when all researchers involved agreed on the classifications.

Finally, the research group examined potential differences between the categories formed for assisted living with 24/7 services and home care. Throughout the analysis, researcher HK-N documented the process in detailed notes.

The credibility of the analysis was supported by collaborative discussions on coding and categories, as well as verification and confirmation of the coding by other researchers, followed by further refinements. Direct quotes from the answers (translated from Finnish to English) are also included in the results. The COREQ checklist [43] was used in the reporting of manuscripts, to ensure the quality of the reporting. The Ethics Board of the Finnish Institute for Health and Welfare has approved the study (THL/1051/6.02.01/2024).

**Table 1:** Analysis example of the formation of categories

Original expression	Subcategory	Generic category	Main category
*“It’s easier to get to know clients in your own team.”* *“Smaller teams have enabled familiar nurses for clients.”*	Nurses and clients get to know each other.	Continuity of care	Benefits of using teams
*”Knowledge of customers’ changing situations is better managed* *“Teams are more up-to-date with the issues of their own team’s clients*	Improved flow of information		
*“The large number of purchase and agency workers*,* makes it difficult for teams to operate.”* *“The challenge at the moment is the huge shortage of nurses*,* which makes teamwork difficult. Teamwork would require some retention of nurses in order to function in the best possible way.”*	Recruitment challenges	Workforce planning	Challenges of using teams
*“If there are absences in teams*,* sometimes nurses have to be transferred to another team.”* *“Staff shortages can be filled by transferring employees to other teams.”*	Transfer workers between teams		

## Results

**Fig. 1:**
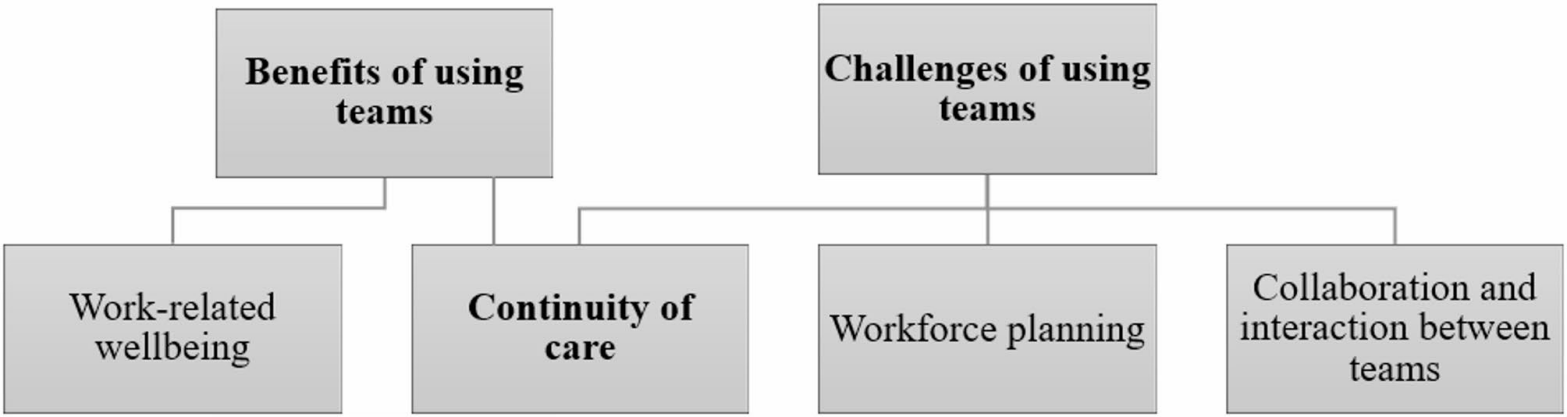
Perceived benefits and challenges of using teams, as experienced by nurse managers in home care and assisted living with 24/7 services

The nurse managers felt that the use of teams had a positive impact on nurses’ well-being at work. Use of teams was seen as both a benefit and a challenge in terms of the continuity of care. Challenges in the use of teams that the nurse managers experienced were workforce planning and collaboration and interaction between teams and professional. (Fig. 1).

### Work-related wellbeing

The improvement in nurses’ coping at work through teamwork was visible to nurse managers in both home care and assisted living with 24/7 services, mainly through a considerable increase in nurses’ work motivation and job satisfaction. In home care, nurse managers perceived that the psychosocial strain experienced by nurses at work had decreased after implementation of a team structure. The nurses’ coping at work was reflected in the nurses’ commitment to work and in addition highlighting the employees’ own competence in both home care and assisted living with 24/7 services.

*Employee self-management in our teams has been increased*,* which has had a positive impact on employee job satisfaction.*

Nurse managers viewed autonomy and self-direction as factors that increased employees’ coping at work. Self-organizing was mainly reflected in the independent planning of nurses’ shifts and work content, which resulted in nurse managers being mainly responsible for the administrative matters of the team and unit. In some units, nurse rotation in different teams was carried out as a continuous activity at the nurses’ request, while in some units of assisted living with 24/7 services, nurses had the opportunity to choose between caring for long-term clients or newer temporary short-term clients. Some of the nurse managers felt that when job rotation between the different teams of the work unit was carried out at the request of the nurses, this increased the nurses’ opportunities to influence their own work, thereby affecting the nurses’ work motivation and job satisfaction.

*One team has more physically demanding clients*,* and the other team has more mentally demanding clients. Efforts have been made to reduce the workload with regular employee rotation. This brings variety to the work and has also been a wish of the employees.*

Being acquainted with the colleagues in the team was seen as an enabling factor for receiving support from the team and for bringing up issues or difficult cases, especially in home care, where nurses mainly work alone. In assisted living with 24/7 services, nurse managers also highlighted the increased sense of community at the workplace and improved nurse manager work as benefits of teamwork. The role of team leaders was also emphasized. A good team leader was seen to take responsibility for the team’s operations and development, which also alleviated the nurse manager’s workload.

Job satisfaction is better when you get to know your colleagues better in teamwork.

*Team spirit is better realized in a smaller group*,* and this significantly increases well-being at work.*

### Continuity of care

The opinions about the effects of teamwork on the continuity of care, were divided among nurse managers. In the responses, successful continuity of care was reflected in better planning, implementation, and evaluation of clients’ care. Furthermore, the number of different clients per nurse was perceived as more reasonable due to teamwork, with smaller teams having their own set of clients in both care settings. This enabled better accounting for the individual needs of the clients, as the caregivers were more familiar with their clients and their relatives. In assisted living with 24/7 services, some nurse managers observed that nurses write more thorough nursing documentation when working in teams.

*The use of teams is positive. Our teams consist of 1–2 nurses as well as practical nurses and care assistants. Everyone takes care of common clients from the perspective of their own expertise. Clients have treatment plans and updated RAI [Resident Assessment Instrument] assessments. The clients’ goals and health challenges are known. There is a weekly team meeting to discuss the current situation of the customers.*

In both care settings, shift planning and nurse work management were seen as having improved because of using teams. Mobility of staff between different teams was identified to ease nurse shortages and help with arranging substitute nurses. In assisted living with 24/7 services, some nurse managers indicated that the unit utilized job rotation in order for the nurses to become familiar with the other teams in advance. In addition, some of the nurse managers felt that teamwork enabled them to have a better opportunity to influence their own work and that the division of duties in the unit had become clearer. The overall pace of work was also perceived as becoming more manageable with the use of teams. In home care, better team-oriented work organization in some units led to a decrease in driving distances.

*Administrative work has been taken away from the teams*,* meaning that nurses are responsible for nursing and practical nurses are responsible for basic nursing.*

Some of the nurse managers felt that collaboration between the unit’s teams improved because of teamwork and that this improvement also affected continuity of care. Especially in assisted living with 24/7 services, however, some of the nurse managers felt that collaboration between teams was seen to have improved through job rotation. On the other hand, some of the nurse managers had noticed that the nurse felt that the work rotation between teams was stressful. Teamwork was seen as contributing to nurses’ self-organizing skills and the creation of common operating practices, and nurses were also seen to take better responsibility for client care. The flow of information in teams was also felt to have improved, even though temporary agency workers and substitutes were seen to occasionally pose challenges to the flow of information.

*The teams also collaborate constantly*,* and the team members regularly go to work shifts in another team. This way*,* whole work unit (not only own team) remains familiar*,* and people are more willing to work in both teams if necessary.”*

*The work atmosphere remains good when you can work with familiar colleagues.*

The use of teams was perceived to further a sense of security for clients and relatives, as a result of the same nurses mainly caring for the same clients. The nurse managers highlighted especially the stabilizing effect of assigned nurses in the treatment of clients with memory disorders. Similarly, the relatives of clients have been content dealing with familiar nurses about their relative’s affairs. In addition, nurse managers felt that matters of the clients were better taken care of when nurses know their clients’ situation and care needs, and consequently notice changes in the client’s state of health more easily. Lastly, nurse managers in home care highlighted clients’ trust in nurses through increased teamwork.

*The teams enable more individualized care for clients*,* as the nurses become familiar to the clients and abnormalities in the client’s condition are noticed faster.*

*Clients with memory disorders are calmer when familiar nurses are mainly caring. When the nurses know the clients*,* the needs of the clients are adapted to the needs of their own team and clientele. Cooperation with relatives is also smooth*,* as the nurses in the team know all clients well.*

However, some of the nurse managers pointed out that nurses’ professional skills may not develop sufficiently when caring for the same clients, and the awareness of what is happening in the other teams narrows if caregivers only work in one team.

In addition, in contrast with previous results on continuity of care, nurse managers also highlighted that assigned nurses may not notice small incremental changes in their own clients’ state of health over time. This was especially relevant in case of possible excessive attachment between the client and the caregiver. Some of the nurse managers pointed out that some nurses feel that changing the clients brings meaningfulness to work, which is not the case when only caring for the same clients. The nurse managers also felt that the nurses’ skills in managing their own work varied significantly. For some nurses, managing their own work seemed challenging, complicating the entire team’s operations.

*In teams*,* assigned nurses causes inflexibility in handling other clients’ affairs*,* blinds operating methods*,* and not enough is known about other customers.*

*Employees’ know-how doesn’t develop if you work in the same team all the time.*

### Workforce planning

Challenges related to workforce planning in units that have implemented teams were highlighted. Resource shortages, recruitment challenges, and the increasing use of temporary agency nurses, all common problems in the care sector, were perceived as significant challenges in both care settings. Nurse managers must consider the total number of temporary and permanent nurses available daily, which means that in case of for example sickness absences, some nurses may not be placed in their own team. From the nurse managers’ point of view, nurses were burdened by moving from one team to another due to sudden absences of employees.

*Teams settle into their own teams so well that they are not very happy to go to another team for a shift. Changing teams is stressful for employees.*

*The challenge in teamwork is the mobility of staff between teams due to staff shortages*,* which nurses find burdensome.*

Ensuring the competence of nurses in the substitute arrangement put a strain on nurse managers. For example, the nurse managers of assisted living with 24/7 services felt that substitutes without a medicine license put a lot of strain on the units, as permanent staff often had to be transferred to another team in order for every team having enough nurses with medicine license. Some substitutes also preferred an employment contract exclusive for a specific team, which was seen as further challenging workforce planning and teamwork.

*We have recurring situations where teams compare each other and the lack of flexibility to move from one team to another. The lack of medical licenses for the personnel is burdensome*,* as well as the unequal distribution of responsibilities between substitutes and permanent employees. In addition*,* employees’ work ethic and initiative vary.*

*Substitutes without a medicine license interfere with the work of teams. Sometimes it is necessary to move employees from one team to another for this reason.*

Lack of resources due to personnel shortages and sudden absences of nurses challenged the continuity of care. In cases of sudden absences, nurse managers tended to primarily transfer nurses between teams in the unit, which according to nurse managers, the nurses find burdensome. Shift work, especially in assisted living with 24/7 services, was also felt to challenge shift planning if some of the nurses mainly worked night shifts. In most cases, night shift nurses were responsible for the clients of several teams. Therefore, some nurse managers considered it necessary for nurses to work in different teams in other shifts in order to get to know all the clients in the unit. According to the nurse managers, nurses found job rotation in different teams stressful in assisted living with 24/7 services, if this was not what the nurses wanted. Interestingly, in some units, the employees preferred job rotation and working in different teams. Nurse managers in home care highlighted the varying number of clients in teams and the suddenly emerging circumstances as significant challenges of using teams. The nurse managers hoped that the nurses would understand the unit’s operations as a broad entity and not see things only from the perspective of their own team. This was especially relevant for when nurses had to be transferred between different teams due to limited workforce resources.

*The flexibility of employees to work in another team if necessary is work-intensive*,* employees do not understand the entirety*,* they just look at things from their own perspective. Employees would also benefit from it (working in another team) by diversifying their own skills.*

In assisted living with 24/7 services, job rotation was also used proactively for sudden absences of nurses. This simultaneously allowed the nurses to become familiar with other teams and their clients. The responses did not expand on the impact of a potential pool of substitutes on the teams’ operations. In some regions, home care visit planning was centralized to the whole wellbeing services county or a municipality, meaning that home care visits were allocated to nurses by a separate unit potentially not familiar with the area. Centralized workforce planning was felt not to take teams into account. Some of the clients may have been from another area and the nurse was not familiar with the clients.

*The division of work between the teams is done centrally in our home care*,* so client visits are not always directed to the right team or personal caregiver*,* even if the customer’s personal caregiver is working.*

### Collaboration and interaction between teams

Poor collaboration between the teams emerged as a challenge in both care settings. While close teamwork was seen to have many benefits, nurse managers also highlighted its negatives. Close collaboration was felt to weaken nurses’ willingness to temporarily move between the teams and to have substitutes in different teams, because some substitutes only wanted to work in a specific team. In addition, close teamwork was seen to increase the competition between teams, which resulted for example in jealousy related to the perceived care intensity of the team’s clients. Sometimes the teams developed their own operating methods, which were not in line with the policies of the care units. If there was no job rotation in place, the use of teams was seen to have a negative impact on the collaboration between the unit’s entire personnel. The flow of information between nurses, especially in the occasional absence of permanent staff, brought challenges to the work. In addition, presence of nurses with varying cultural backgrounds was sometimes seen as causing challenges in teams due to language barriers and misunderstandings.

*The challenge is that teams and their ways of working may diverge too much. In this case*,* sticking to uniform operating methods and culture is challenging and poses challenges for leadership. It requires management to have the means to ensure that employees remain prepared and have a positive attitude to work in another team as well*,* so that job rotation remains positive and beneficial for everyone.*

*The challenge with teams is that they develop a wide range of operating methods*,* the appropriateness of which has to be assessed.*

Another challenge was the formation of close cliques between teams, which did not interact with each other, sometimes to the detriment of quality of care. According to the nurse managers, the nurses did not dare to interfere in the work of another team if they noticed care work conflicting with the clients’ care and service plans. Nurse attitudes, motivation and negativity towards change were also related to successful collaboration and interaction within teams and across team boundaries.

*The employees’ sense of belonging at the level of the entire unit must be ensured somehow*,* so that teamwork is not carried out in a way that complicates the unit’s operations as a whole.*

*You don’t necessarily interfere with someone else’s work*,* even if you notice that the other person is acting differently from the treatment plan.*

*If cooperation between team members does not go well*,* it negatively affects the entire team’s operations and the well-being of the individuals in the team. At worst*,* this is also reflected in the success of the clients’ treatment.*

## Discussion

First, it is important to highlight that there were no fully self-directed teams in this study. In this study, nurses refer to both practical and registered nurses, as survey respondents mainly used the word “nurse” in their answers. In most cases, the teams often consisted of one or two registered nurses acting as team leaders. Team leaders often had medical responsibility for the teams’ clients, often acted as experts in their own team for practical nurses and were responsible for the day-to-day management of the teams. In addition, the team leader acted as a link between the nurses and the nurse manager. The nurse manager was often responsible for the team’s administrative matters, such as recruitment and workforce planning. The nurses were allowed to participate in the planning of shifts and holidays, but these were finalized by the nurse managers.

The aim of this study was to describe the benefits and challenges of teams, as experienced by nurse managers in services for the older people. The results of the study show that the use of teams has potential to improve nurses’ wellbeing at work and he quality of care, but it also involves challenges, especially in workforce planning. This study showed that teams might have a positive effect on the psychosocial strain experienced by caregivers and has been somewhat successful in further engaging nurses in their work. Previous studies have shown that self-organized teams have mainly positive effects on worker well-being and quality of care [13–15].

As noted, this study was not exploring only the self-organized teams, but all kinds of teamwork. Autonomy of the teams may vary strongly, but it seems, that more autonomy leads to better worker well-being [44]. Implementing more self-organized teamwork is not a straightforward task, which automatically leads to better worker well-being. There are also several barriers in implementing self-organized teamwork, such as management or organizational culture [12, 14, 45]. Harkema and colleagues [46] showed that several elements of organizational structure hinder self-organized team success. For example, too specialized, centralized, or formalized teams do not lead to better worker wellbeing. Also, very low level of these characteristics decreases the success of teamwork. In addition, this study showed that there are several factors affecting teamwork. The work was organized in teams, but still the work organization was centralized and the manager led more than one team. On some occasions, the employees had to work outside their own team. However, from the managers’ perspective, too high commitment to the nurses’ own teams and teams’ greatly varying working methods made it difficult to work in other teams when needed. Psychosocial workload factors have previously been found to be the most significant determinants of nurse turnover [3]. In addition, better work division and team autonomy might reduce perceived stress and time pressure of nurses [44].

Our results also indicate that primary nursing could be better implemented with the use of teams, which was seen to have a positive effect on the quality and continuity of care. Lack of cooperation between nursing staff has been identified as one major obstacle to achieving continuity of care for clients [47]. Working in a team was seen to make it easier to get to know colleagues and thus make it easier to bring up potential issues. Nurse managers also felt that nurses were more self-directed in teams and that employees took better responsibility for the care of clients. These results are in line with previous studies, where autonomy has been found to increase nurse satisfaction and engagement [15, 48].

Nurse managers had complex and sometimes clashing views on the benefits and challenges of teams. In the present study, negative views of teamwork and increased autonomy were also brought up. For instance, some teams developed unique ways of working, which challenged common rules and practices. The different modes of operation in the teams were seen to make it difficult for nurses to rotate between different teams. It can be brought into the discussion whether the different working methods in teams is a challenge from the point of view of the care organization or teams. However, the teamwork model requires close collaboration between team members to function. It is also important to pay attention to collaboration between teams and find ways to reduce potential competition between teams. The teamwork model also does not materialize if team members must constantly work in different teams due to staff shortages [18, 19]. Research has also found a positive relationship between team cohesion and performance [49], which can be influenced especially by paying attention to cooperative satisfaction and consistency of affection [50]. On the other hand, the search for consensus can increase with team cohesion, and consequently weaken team performance [51].

There were also conflicting issues regarding the collaboration between teams. In some units, the collaboration between teams was felt to have improved through job rotation in different teams. In other units, collaboration between nurses was felt to have deteriorated at the unit level, as nurses did not rotate between different teams, and instead became more familiarized with members of their own team. As increased collaboration has been associated with lower perceived job demands and higher job control [44], the potential positive and negative effects of teamwork on collaboration are important. Consequently, from the view of collaboration within teams, teamwork might especially benefit units with more permanent staff and less turnover.

Some of the challenges with the teams could be attributed to the lack of real autonomy of teams and the rotation of nurses between teams, which was justified by external reasons, such as centralized planning system in home care or too limited skills of temporary workers. Rotation was also justified by the low development of nurse skills, especially when clients are not changing. In previous research, self-organizing teamwork has been found to benefit nurses working in home care and assisted living with 24/7 services, but implementing self-organizing in teams also requires significant changes in management and successful team building [14].

The present study showed that permanently working nurses could not always be placed in their own team, as managers had to ensure a sufficient number of skilled nurses in the teams. A significant work management issue highlighted in the study was the nurses lack of licenses to administer medications, which resulted in the unnecessary moving of nurses between the different teams to ensure sufficient competence of pharmacotherapy. As such, secondary factors, e.g., the completion and validity of nurses’ licenses to administer medications, might arise as surprising components of alleviating the workload and substitute arrangements, and ultimately enabling functional teamwork.

On the one hand, the results highlighted the development of nurses’ professional competence if the nurses only take care of the same clients continuously. Getting to know clients better and having more autonomy was seen as increasing worker wellbeing. On the other hand, nurse managers felt that rotation between different teams helped develop professional skills, and also had an effect on coping and wellbeing at work if the rotation was implemented at the request of nurses. Job rotation has been seen to be positively linked to job satisfaction and organizational commitment [52]. There were also conflicting views among nurse managers on whether nurses better perceive changes in a client’s health by treating only the same clients or whether nurses need to treat different clients to different degrees. Similar results emerge from a study by Corneliusson and colleagues [53], in which new nurses were seen to notice subtle changes better the client’s health, which were in danger of otherwise going unnoticed by the assigned nurses. In addition, job rotation was seen to enable permanent personnel to work with different clients, which increased job satisfaction. However, research has also shown that nurses have lower job satisfaction the more they are required to take care of unfamiliar clients [16].

Sufficient training and resources for nurses to provide high-quality client care have been found to promote nurses’ commitment to work [54]. Nurses have felt that further studies, such as courses and specializations, support professionalism [55]. However, it remains to be considered whether continuing education alone will be enough to maintain nurses’ professional competence in teamwork to a sufficient extent if nurses are not able to put their skills to practical use. Our results indicate that when using the teamwork model, special attention should be paid to maintaining and developing the professional skills of nurses. However, continuous transferring of nurses between teams with the goal of maintaining professional skills is not a teamwork compatible solution.

Another result was the identified discrepancy between how much responsibility nurse managers expect nurses to take in teams and how much responsibility they actually take. Some nurse managers felt that nurses were reluctant to work as a team and did not take sufficient responsibility for working in a team. However, it remains unclear whether the nurse managers and nurses have a common understanding on the level of independence nurses should operate on in the teamwork model. Ruotsalainen and colleagues [23] found that for teams to operate in a fully self-organizing manner, managers would need to give teams full autonomy. However, in this study, we were unable to distinguish the level of autonomy teams had. Nurse managers’ views on the teamwork model can have an impact on the functionality of the teamwork model. If the nurse manager sees the work of the unit only as a whole, rather than the work of independent teams, sufficient support for teamwork may not be given. This might limit the potential positive effects of teamwork, and as such, it is essential to ensure that both the management and the nurses are aligned when implementing teams.

When examining teamwork and self-directed teamwork in the care sector as part of the broader field of organizational research, self-directed teams appear to have become increasingly common in other sectors. Although the care sector differs from many other industries in terms of ethical requirements and regulatory frameworks, self-management can also be effective in this context—provided that organizational structures and culture support the autonomy of employees and teams [56]. 

This study revealed that teams usually do not have completely independent responsibility for the team’s activities, but the autonomy of nurses in matters concerning the team varies from unit to unit. As a rule, managers were responsible for workforce planning, even though working time autonomy was utilized. In the original Buurtzorg model, on the other hand, teams manage and organize their own work holistically in terms of client work, recruitment, and workforce planning [18, 19]. However, full independence of teams might not always be necessary, as studies have also shown that for example coaching leadership has a positive effect on nurses’ wellbeing at work and the quality of nursing [23, 57]. However, this limits the transferability of both the care models (such as Buurtzorg) and the research concerning them, if fully self-directed teams are not possible. Consequently, further research on the effects of more limited forms of team autonomy in care work is required.

In home care, one significant problem was that shift planning and enterprise resource planning (ERP) systems often cannot or do not take teams into account. While ERP systems have been able to optimize care visits, increasing productivity and mitigating peak hours [58], they might have simultaneously narrowed the influence of teams. This might have led to increased dissatisfaction with work among nurses [7].

The long-term effects of teamwork need to be studied holistically from the perspective of nurse managers, nurses, and clients. In this study, we examined the perceived challenges and benefits of the nurse managers. Continuity of care and improved work-related wellbeing of nurses were seen as the biggest advantages of teamwork, and the availability of workforce planning as a challenge. Further research is needed on the integration of ERP systems and self-organized teams. In addition, it is necessary to investigate how personnel resourcing and team workforce planning could be supported and optimized from the perspective of quality of care, work efficiency, and job satisfaction. It’s also good to point out that there doesn’t necessarily exist a one-size-fits-all nursing model [59], and instead the working methods and care models should be planned in close collaboration with the care personnel while taking the needs of the clients into account.

International studies also show that different models of home and community care produce varying outcomes depending on structural emphases, and that adapting care models to specific goals is central to their effectiveness [60], which aligns with the findings of our research. However, in the context of assisted living with 24/7 services and home care in Finland, variation in team structures between units may influence how closely these models resemble international solutions. This structural variation may also affect the generalizability of the results and highlights the need for comparative research that situates our findings within the broader international care discourse. It should be noted, however, that this study did not focus on a specific team or care model, but rather on experiences of different team structures.

## Limitations

The study has some limitations. First, the results of the study concern the use of teams. The answers are based on the respondents’ own definitions of a team, which may impact the consistency of the results. However, the survey instructions defined teams in the survey, and they might for example refer to fully self-directed teams, partly autonomous teams, or merely floors or wings of a physical building where the unit is located. As a result, the findings are not fully comparable to the Dutch Buurtzorg model, as some units had implemented rotation of nurses between the teams, unlike the Buurtzorg model, where employees work in independent teams [29, 30]. Importantly, most organizations have not implemented fully self-organized team models, meaning that decision making authority in the teams is often limited.

The number of respondents in the nationwide survey was large (*n* = 910) and the response rates were high for both care settings. It should be noted that the answers were relatively short, consisting of individual words or short sentences. More in-depth answers would have been obtained, for example, by conducting interviews. To increase the credibility of the study, the analysis was discussed in the research group in order to achieve a common understanding of the results.

## Conclusion

Similar benefits and challenges related to the teamwork model were shown in both home care and assisted living with 24/7 services. According to nurse managers, the use of teams and teamwork can be used to promote the nurses’ wellbeing at work, reduce psychosocial burden experienced by nurses, and improve the quality and continuity of care. Implementing a teamwork model in an organization requires work, and it is important to transparently discuss the exact roles, responsibilities, and obligations for both the nurse managers and the nurses. Teamwork is challenged by temporary agency workers, the development of nurses’ professional skills, and collaboration between teams. The results can be utilized in developing nurses’ wellbeing at work and the quality of care in services for older people, which can improve the care sector’s attraction and retention. Future research should examine the best ways to integrate new employees and substitutes into the teams (e.g. different induction practices, mentoring) and how to maintain cooperation between teams (e.g. leadership, culture, common team meetings). In home care, further research is needed on the coordination of ERP systems and teams.

## Data Availability

The datasets generated and analyzed during the current study are not publicly available, as the survey is mandated by law and conducted for administrative purposes, but are available from the corresponding author on reasonable request. Some of the results are reported by Kehusmaa and colleagues, which has been cited in the manuscript [38]. https://www.julkari.fi/bitstream/handle/10024/150082/URN_ISBN_978-952-408-324-9.pdf?sequence=1.

## Abbreviations

ERP: Enterprise resource planning
JD-R: Job Demands-Resources model
